# Prognostic significance of preoperative neutrophil-to-lymphocyte ratio in lung carcinoid patients after receiving curative surgery. A multicentre study

**DOI:** 10.3389/fonc.2025.1585433

**Published:** 2025-06-10

**Authors:** Nicola Tamburini, Beatrice Aramini, Ilaria Potenza, Francesco Bagolini, Danila Azzolina, Fares Shamshoum, Federico Rea, Franco Stella, Giampiero Dolci, Enrica Pellizzer, Pio Maniscalco, Andrea Dell’Amore

**Affiliations:** ^1^ Department of Thoracic Surgery, Sant’Anna University Hospital, Ferrara, Italy; ^2^ Thoracic Surgery Unit, Department of Medical and Surgical Sciences-DIMEC of the Alma Mater Studiorum, University of Bologna, G.B. Morgagni-L. Pierantoni Hospital, Forlì, Italy; ^3^ Department of Environmental and Preventive Science, University of Ferrara, Ferrara, Italy; ^4^ Department of Cardiothoracic Surgery and Vascular Sciences, Padua University Hospital, University of Padua, Padua, Italy

**Keywords:** neutrophil-to-lymphocyte ratio (NLR), pulmonary carcinoid, survival & prognosis, non-small cell lung cancer (NSCLC), serum c-reactive protein

## Abstract

**Background:**

Recent evidence suggests that inflammation is relevant to carcinogenesis and tumor progression. Biomarkers of the inflammatory response are increasingly regarded as valuable prognostic indicators for enhancing predictive accuracy in non-small cell lung cancer (NSCLC). Nonetheless, the applicability of these measures in patients with pulmonary carcinoid remains uncertain.

**Objectives:**

The primary outcome of this study was to evaluate the prognostic impact on Overall Survival (OS) of the preoperative neutrophil-to-lymphocyte ratio (NLR), platelet-to-lymphocyte ratio (PLR), and serum C-reactive protein (CRP) in pulmonary carcinoid after complete surgical resection.

**Methods:**

We retrospectively evaluated data from 267 patients who underwent surgery with curative intent for pulmonary carcinoid tumors between January 2010 and December 2020. Peripheral blood samples were collected preoperatively during routine preoperative tests. The univariable-unadjusted and the Inverse of Probability Weight (IPW) propensity score (PS) adjusted Cox regression models are reported to assess the association between inflammatory biomarkers and outcomes.

**Results:**

The median follow-up duration after surgical resection was four years. Elevated NLR was the only biomarker significantly associated with worse overall survival (OS). The significant association between NLR and OS is evidenced after adjusting for potential confounders using IPW.

**Conclusions:**

This study demonstrated a significant association between the NLR in blood samples of carcinoid patients and their survival outcomes.

## Introduction

Lung neuroendocrine tumors (NETs) encompass a range of malignancies, from highly aggressive forms like large cell neuroendocrine carcinoma (LCNEC) and small cell lung cancer (SCLC) to the typically slow-growing pulmonary carcinoid (PC) tumors, which can be either typical or atypical. PCs are sometimes discovered incidentally and often follow an indolent course. Although they represent only 2% of all lung cancer cases, PCs account for approximately 25% of all NETs (1, 2).

The incidence of all NETs is increasing: according to a Surveillance, Epidemiology, and End Results (SEER) analysis from 1973 to 2012, the incidence of NETs grew 6.4-fold between 1973 and 2012 (3). This rise was most significant in PCs, which had the highest incidence.

Surgery is the recommended treatment for localized PCs. It has demonstrated good five-year survival rates, with 94% for TC and 67% for AC (4, 5). Due to the disease’s variable prognosis, it is essential to search for potentially helpful biomarkers for the early diagnosis and prognosis of PC patients.

In recent years, numerous studies have demonstrated a strong association between inflammation and the initiation and progression of malignant tumors. Furthermore, inflammatory marker levels have been shown to influence the prognosis of patients with malignancies (6, 7). Several intricate mechanisms lead to the progression of a tumor from its genesis to promotion and metastasis. These mechanisms include aberrant activation of inflammatory cytokines, abnormal proliferation of malignant cells, immune system dysfunction in the host, and damage to DNA (8).

The prognosis of cancer patients may be predicted by circulatory biomarkers, which indicate the degree of inflammation. Among peripheral blood biomarkers of inflammation, the neutrophil-to-lymphocyte ratio (NLR) has become a more significant prognostic indicator in patients with pancreatic cancer, gastric cancer, hepatocellular carcinoma, breast cancer, and soft tissue sarcomas. Some studies have identified an association between the platelet-to-lymphocyte ratio (PLR) and lymphocyte-to-monocyte ratio (LMR) with unfavorable outcomes in various malignant tumors (9–13). Nevertheless, the utility of these markers in predicting the prognosis of patients with pulmonary carcinoids remains debatable.

We hypothesized that NLR, PLR, and CRP may be potential indicators in patients affected by pulmonary carcinoid tumors. In this work, we sought to evaluate and analyze the prognostic effects of these biomarkers in patients with pulmonary carcinoid undergoing surgical resection, to develop prognostic factors to improve outcome prediction for pulmonary carcinoid patients.

## Patients and methods

The medical records of 267 newly diagnosed pulmonary carcinoid patients were analyzed. Surgery was performed in three different thoracic departments between January 2010 and June 2020. All patients underwent standard diagnostic laboratory evaluations, including electrocardiography, spirometry, and arterial blood gas analysis. Additionally, chest imaging was conducted through computed tomography (CT) scans. Pulmonary carcinoid was diagnosed based on histological evidence and categorized using the 8th edition of the TNM-UICC/AJCC classification (14).

The present study was performed according to the principles outlined in the Declaration of Helsinki for human experimentation, and local ethics committees approved the protocol. Blood samples were collected within ten days before and after the surgical intervention. Pre-treatment NLR, PLR and CRP were calculated from peripheral blood cell count. The NLR was defined as the absolute neutrophil count divided by the absolute lymphocyte count and categorized into two groups (13); similarly, PLR was defined as the absolute platelet count divided by the absolute lymphocyte count and categorized into two groups. We considered the cutoff of 3 for NLR, dividing the cohort into subjects with an NLR ratio greater than three and those with a ratio less than 3.

The inclusion criteria were: (1) All patients with pulmonary carcinoid tumors undergoing lung resection with radical intent. (2) No infection, hyperpyrexia, and history of hematological disease.

Each patient was followed up regularly until December 2023 or until death (every six months for the first two years, then every six months for the next five). Every consultation included a physical examination, laboratory testing, and a radiologic examination. Overall survival (OS) was determined from the date of surgery to the date of death or final follow-up.

## Statistical analysis

### Data description

Descriptive statistics summarize baseline characteristics, with continuous variables presented as medians and interquartile ranges (IQRs) and categorical variables as frequencies and percentages. We first performed descriptive univariable analyses to explore associations between baseline variables and overall survival. Categorical comparisons were reported as hazard ratios (HRs) with 95% confidence intervals using unadjusted Cox proportional hazards models. The descriptive cut point for the biomarker variable is derived from the descriptive table. *In our analysis, we initially adopted an NLR cutoff of 3, based on previously published literature across several tumor types, including lung cancer, where a threshold of NLR ≥3 has consistently been associated with poorer prognosis (e.g., [10.1111/1759-7714.14646])* (15–17)*. For PLR ([10.1007/s13304-023-01669-3]) and CRP ([10.1177/1479972309104660]), the thresholds of 155 and 2.7, respectively, have been derived from prior literature and are interpreted accordingly* (18, 19).

### Propensity-adjusted models

To further assess prognostic significance, both unadjusted and adjusted models were estimated, treating NLR, PLR, and CRP as continuous variables. The adjusted models were fitted using inverse probability weighting (IPW) based on a propensity score to control confounding. The propensity score was calculated by accounting for age, sex, ASA score, and the Charlson comorbidity index to balance other confounders affecting the survival outcome behind NLR, PLR, and LMR. The selection of confounding factors was derived from clinical judgement.

Weighted Cox models with inverse probability weighting (IPW) were employed to adjust for confounding. Restricted cubic splines were applied to assess the nonlinear effects of continuous predictors and identify possible change points in the risk pattern. The propensity-adjusted and unadjusted estimates have been reported with marginal effects estimation for reporting unit increase effects in hazard ratios for continuous biomarker variables.

Model predictive performance was validated using bootstrapping with 1,000 iterations, and the C Index was calculated to evaluate discrimination. Analyses were conducted by considering the R 3.4.2 (20) System.

## Results

This study analyzed data from a total of 267 patients. Most of these patients were female (147, 66%), with a median age of 60.0 years. The median follow-up duration was approximately four years. Demographic data and clinicopathological characteristics of the patients are summarized in **Table 1**. Fifteen patients (6%) died during the follow-up period, ten of them were male (67%). Male patients have a four-and-a-half times greater risk of mortality than female patients. The hazard ratio analysis for age indicates that each additional year is associated with a hazard ratio of 1.09, suggesting a 9% increase in mortality risk per year. We divided the ASA score into two categories: patients with a score of 0–2 and those with a score of 3-4. While patients with ASA scores of 3–4 exhibit a trend toward poorer prognosis, this association does not achieve statistical significance (p=0.059, HR 3.27). In contrast, the Charlson comorbidity index shows a more pronounced effect (p=0.001, HR 13.3). Each additional point on the Charlson index, which reflects the patient’s overall comorbidity burden, is associated with an increased mortality risk, indicating higher mortality among more frail individuals. Furthermore, patients undergoing surgery for atypical carcinoids showed a poorer survival (p= 0.003, HR 4.65). The focus of our study is the investigation of the NL ratio. We consider the cutoff of 3, thus dividing the cohort into subjects with an NL ratio greater than three and those with less than 3. The univariable estimates in **Table 1** evidenced that the NL ratio and other dicotomized biomarkers (PLR and CPR) did not show a statistically significant effect on Survival.

**Table 1:** Patient characteristics.

Variables	Total	No death	Death	*Unadjusted-Univariable HR*	p
	N= 267	N=252	N=15		
Patients Characteristics					
Sex • Male	90 (33.7%)	80 (31.7%)	10 (66.7%)	4.67 [1.59;13.7]	0.005
• Female	177 (66.3%)	172 (68.3%)	5 (33.3%)	Ref.	Ref.
Age, years					
• Mean ± SD	59.0 (14.4)	58.3 (14.4)	70.5 (8.59)	1.09 [1.03;1.16]	0.002
ASA Score					
• 0-2	154 (67.5%)	150 (69.1%)	4 (36.4%)	Ref.	Ref.
• 3-4	71 (35.7%)	64 (34.0%)	7 (63.6%)	3.69 [1.08;12.6]	0.038
Charlson comorbidity index (mean)	4.01 (1.75%)	3.79 (1.62%)	6.00 (1.57%)	13.3 [2.92;60.4]	0.001
Endocrine syndromes	5 (2.23%)	5 (2.39%)	0 (0.00%)	*NE	0.998
COPD	12 (4.72%)	11 (4.58%)	1 (7.14%)	1.38 [0.18;10.6]	0.759
Tumor and Operative Variables					
Complete resection	259 (97.4%)	246 (97.6%)	13 (92.9%)	0.49 [0.06;3.80]	0.497
Histology • Typical carcinoid	200 (74.9%)	193 (76.6%)	7 (46.7%)	Ref.	Ref.
• Atypical carcinoid	67 (25.1%)	59 (23.4%)	8 (53.3%)	4.00 [1.45;11.1]	0.008
Tumor location • Central	65 (24.3%)	59 (23.4%)	6 (40.0%)	Ref.	Ref.
• Peripheral	202 (75.7%)	193 (76.6%)	9 (60.0%)	0.51 [0.18;1.44]	0.206
TNM stage • I	223 (83.5%)	213 (84.5%)	10 (66.7%)	Ref.	Ref.
• II	23 (8.61%)	21 (8.33%)	2 (13.3%)	2.57 [0.56;11.8]	0.225
• III	19 (7.12%)	17 (6.75%)	2 (13.3%)	2.47 [0.54;11.3]	0.243
• IV	2 (0.75%)	1 (0.40%)	1 (6.67%)	8.50 [1.05;68.7]	0.045
Extent of resection • Wedge	26 (9.74%)	25 (9.92%)	1 (6.67%)	0.65 [0.08;4.93]	0.674
• Lobectomy	183 (68.5%)	172 (68.3%)	11 (73.3%)	1.18 [0.38;3.73]	0.774
• Segmentectomy	31 (11.6%)	29 (11.5%)	2 (13.3%)	1.69 [0.38;7.54]	0.491
• Pneumonectomy	6 (2.25%)	5 (1.98%)	1 (6.67%)	1.93 [0.25;14.8]	0.527
• Bronchial sleeve	35 (13.1%)	35 (13.9%)	0 (0.00%)	*NE	0.998
Biomarker-Based Variable					
NLR ratio • <3	154 (57.7%)	146 (57.9%)	8 (53.3%)	Ref.	Ref.
• >=3	113 (42.3%)	106 (42.1%)	7 (46.7%)	1.60 [0.57;4.48]	0.371
PLR ratio • <155	162 (60.7%)	152 (60.3%)	10 (66.7%)	Ref.	Ref.
• >=155	105 (39.3%)	100 (39.7%)	5 (33.3%)	0.94 [0.32;2.78]	0.915
CPR (mg/dl) • <2.7	66 (31.3%)	61 (30.5%)	5 (45.5%)	Ref.	Ref.
• >= 2.7	145 (68.7%)	139 (69.5%)	6 (54.5%)	0.82 [0.25;2.71]	0.740

*NE, Not Estimable.

Data are reported as median and IQR for quantitative variables, and absolute and relative frequencies for qualitative variables. The univariable Cox HR (Hazard Patio) has been reported with 95% CI and P-Values.

**Figure 1:**
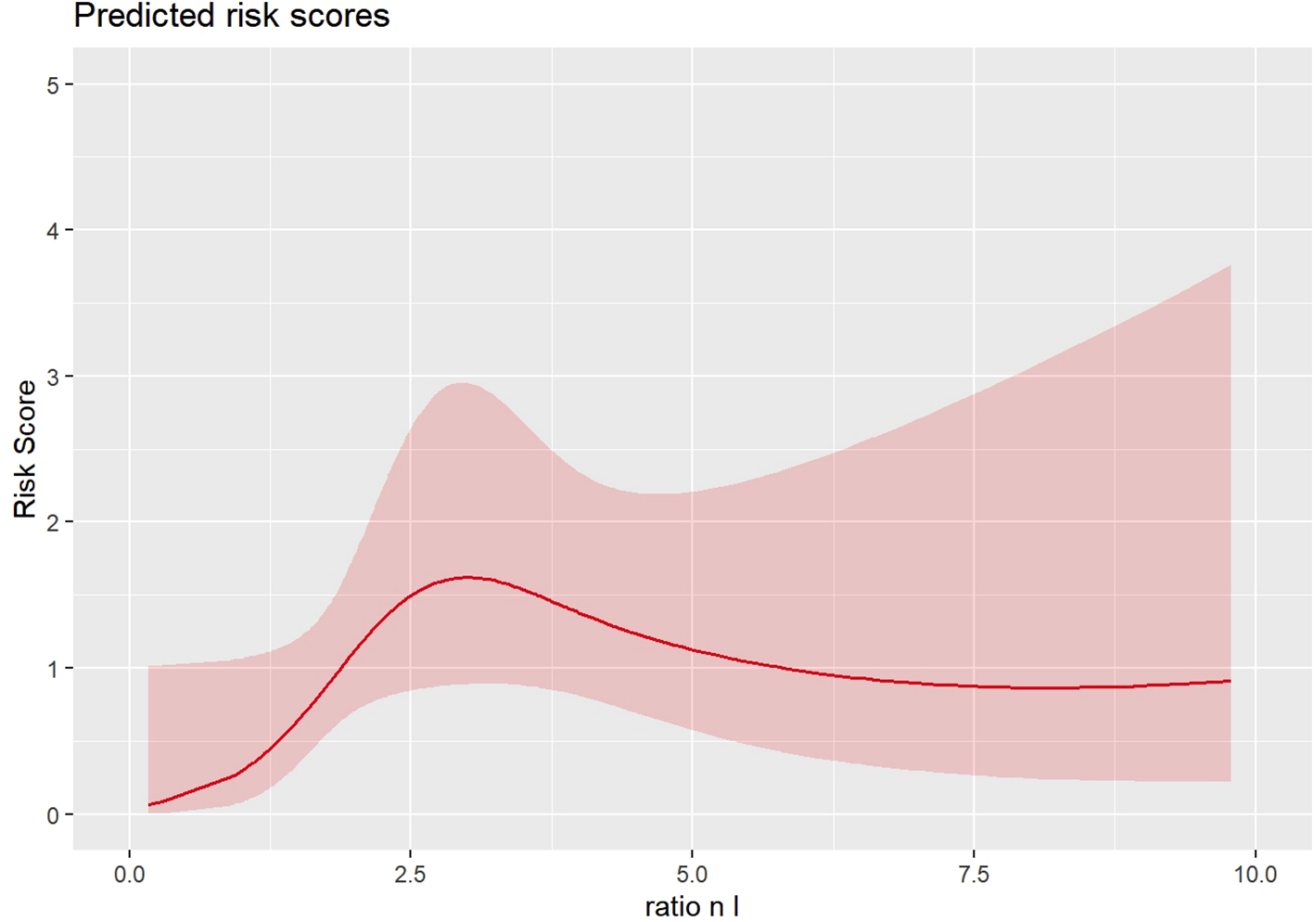
Propensity score-adjusted model.

In the propensity score-adjusted model (**Figure 1**, **Table 2**), we observed an increase in the risk of death until an NLR equal to 3, corresponding to a change in the slope of the risk curve. When the NL ratio decreases from 2 to 1, the propensity-adjusted model evidenced prognostic value of NLR (HR 1.05, 95% CI [1.006–1.09], p=0.024). No significant effects are reported in unadjusted models considering continuous biomarkers as a covariate (**Table 2**).

**Table 2:** Cox models with inverse probability weighting (IPW) hazard ratios (HR) and Model predictive performance validated with bootstrapping with 1,000 iterations, and the C Index with 95% CI.

Model	Adjusted HR 95%CI	P-Value	Unadjusted HR 95%CI	P Value	C index
NLR	1.05 [1.006-1.09]	0.024	1.05 [0.99-1.11]	0.08	0.66 [0.51-0.8]
PLR	0.99 [0.96-1.01]	0.53	0.99 [0.95-1.03]	0.66	0.51 [0.61-0.56]
LMR	0.98 [0.89-1.04]	0.47	0.98 [0.91-1.04]	0.46	0.63 [0.51-0.78]

Propensity score-adjusted and unadjusted estimates have been reported. Restricted cubic splines estimation has been performed to assess the nonlinear effects.

The propensity balance diagnostic was inspected via a balancing plot, which reveals a suitable balance across covariates. Patients with lower NLR scores had a significantly longer survival probability compared to those with higher scores.

## Discussion

In our prognosis analysis in pulmonary carcinoid patients, the adjusted model indicates that NLR was associated with overall survival (OS). In contrast, CPR and PLR showed no significant association with OS.

The host immune system has a crucial role in determining the outcome of cancer (21). Recently, white cell ratio biomarkers—like NLR—have emerged as a possible simple index for clinicians to to classify patients with neoplastic disease risk. It is unclear what mechanisms underlie the association between elevated NLR and poor survival in cancer. Tumor-infiltrating immune cells, which have the ability to regulate tumor growth, may help to explain this connection. Specifically, it is believed that growth factors and chemokines-mediated tumor-associated neutrophils (TANs) stimulate angiogenesis and accelerate the development of tumors (22). Lymphocytes are immune cells that are primarily responsible for the body’s protective effector immunological response and antitumor response. In other words, a decline in circulating lymphocytes signifies a lowering of immune surveillance, which permits the development of tumors (23). Therefore, a high NLR may indicate inadequate antitumor immunity and a favorable environment for tumor development and metastasis.

An increased NLR has been demonstrated to be related with lower overall survival in patients with a range of specific tumor types (24–31). However, no study has analyzed a cohort of patients affected by carcinoid tumors. The present study suggests that a preoperative elevated NLR of 3 or higher was significantly associated with poor outcome in pulmonary carcinoids undergoing surgical resection. Therefore, NLR can be used in counseling about predicted outcomes as well as in the development of new clinical trial designs based on systemic inflammatory indicators. This analysis can be utilized in real-time clinical practice, in addiction to other risk stratification items, to assist patients and healthcare practitioners in making a choice about whether to definine the postoperative surveillance intensity.

Different values of NLR, with different methods, in different populations (cancerous or not) are cited in the literature. And finally, no universal value is currently available highlighting the need to establish a consistent optimal cutoff value for PLR, NLR, and LWR. The choice of patients, their baseline features, and the accessibility of the clinical data from each study may all be indicators of this. Other studies confirmed our findings (32, 33). Furthermore, most studies did not investigate the predictive importance of serial NLR monitoring in comparison to baseline NLR alone.

In this study we did not find a significant correlation between PLR and CRP levels with survival. The relationship between PLR, CRP, and other inflammatory markers is complex and depends on the specific context and disease. While some studies demonstrate a strong association, others reveal no significant correlation, highlighting the need for further research to fully understand the role of these markers in various health conditions (34, 35). In a Meta-analysis analyzing the prognostic value of PLR in lung Cancer, PLR had significantly prognostic value for overall survival, but not for progress-free survival (36, 37). From a biological perspective, pulmonary carcinoids are well-differentiated, slow-growing neuroendocrine tumors with distinct tumor biology compared to more aggressive carcinomas. The degree of systemic inflammation typically associated with high-grade tumors may be absent or minimal in these cases. Therefore, systemic inflammatory markers like PLR and CRP may lack prognostic sensitivity in this context. Furthermore, the relatively indolent course and lower tumor burden in typical carcinoid patients may result in weaker or more variable activation of inflammatory pathways. Consequently, these findings will need validation in future research.

Current guidelines do not recommend adjuvant treatment based on preoperative inflammatory markers. Likewise, the routine administration of adjuvant therapies did not appear to improve survival in patients who underwent complete resection (38). Given the lack of current guideline-directed adjuvant treatment in resected pulmonary carcinoid, we expect that NLR biomarker will help to identify high-risk patients who may be enrolled in future clinical trials or targeted for innovative therapeutic options. Additionally, combining NLR with other biomarkers such as chromogranin A, circulating tumor cells (CTCs), or transcriptome profiles might improve prognostic accuracy in specific patient populations, notably in gastrointestinal malignancies and NSCLC (39, 40). An integrated method may produce more powerful prediction tools.

Despite its strengths, our study also had some limitations. First, the number of observed events (15 deaths among 267 patients) is relatively low, which limits the statistical power of multivariate survival analyses and may reduce the robustness and generalizability of the model estimates. Although we used a parsimonious set of covariates and applied a propensity score-based IPW approach to mitigate overfitting, the risk of unstable hazard ratio estimates remains. Furthermore, other inflammatory indicators, such as procalcitonin levels, have been investigated for their predictive significance in cancer patients, but are not tested in our study. Another weakness of the study is that multivariate survival analysis has limited statistical power because there were only 15 deaths in a cohort of 267 people. Another limitation is the lack of data on recurrence and disease-specific survival, which could have provided more nuanced insights into tumor progression. Additionally, no patients received adjuvant therapy, limiting the ability to evaluate potential treatment-related confounding. However, this treatment homogeneity strengthens the internal validity of our survival analysis by removing post-surgical therapeutic variability.

Despite these limitations, in the current study preoperative NLR correlated with OS, indicating that NLR might be utilized to identify patients who should/should not receive adjuvant therapy. Future prospective studies might validate NLR as a predictive biomarker and support its clinical application.

## Data Availability

The data analyzed in this study is subject to the following licenses/restrictions: only authors. Requests to access these datasets should be directed to tamburininicola@gmail.com.
